# PS-37 - CHICKEN OR EGG? MULTIDISCIPLINARY COLLABORATION REVEALS A NOVEL EXPOSURE IN A SMALL PUB SALMONELLA OUTBREAK

**DOI:** 10.1093/pubmed/fdag046.073

**Published:** 2026-07-09

**Authors:** Mrs Caroline Pollard, Mrs Nika Raphaely, Mrs Emilie Bellhouse, Miss Ellie Mackey, Miss Grace King, Mrs Charlotte Anderson, Mrs Kirsten Mueller, Mrs Caroline Willis, Mrs Ann Hoban, Mrs Marie Chatterway, Miss Anna Murasak, Mr Tim Wyatt, Mrs Lauren Dyer

**Affiliations:** UK Health Security Agency; UK Health Security Agency; UK Health Security Agency; UK Health Security Agency; UK Health Security Agency; UK Health Security Agency; UK Health Security Agency; UK Health Security Agency; UK Health Security Agency; UK Health Security Agency; Chichester District Council; Chichester District Council; Chichester District Council

## Abstract

**Background:**

Ten days after a small pub’s Sunday lunch service, the local Health Protection Team was notified by Environmental Health (EH) of gastrointestinal illness affecting multiple diners (eventually 17 of 53). Illness was unusually severe, with four individuals attending hospital. *Salmonella* was isolated from clinical samples. Early reports from diners and initial EH findings suggested multiple potential exposures. Delayed reporting meant no food samples were available to test.

**Objectives:**

To identify the source of infection, prevent further cases, and contribute to national surveillance through a comprehensive outbreak investigation.

**Methods:**

EH officers conducted a detailed inspection, including review of food handling practices and environmental sampling. An Incident Management Team (IMT) was convened. Whole genome sequencing (WGS) was undertaken on clinical isolates. The Field Epidemiology Service undertook a retrospective cohort study using an online questionnaire sent to diners and staff present on the exposure date.

**Results:**

Inspection identified imported Polish eggs stored in the pub kitchen. EH’s initial concerns focused on sous vide temperatures in beef preparation. Food, Water and Environmental testing found unsatisfactory levels of *E. coli* and *Enterobacteriaceae* on kitchen surfaces and in equipment. WGS demonstrated identical single nucleotide polymorphism profiles across four human isolates, consistent with a strain previously associated with imported Polish chickens, but not previously with eggs. Complete responses were received from 26 individuals, of whom 15 met the case definition. Over half sought healthcare and 40% required time off work or education. Only one menu item—the crab profiterole snack bound with raw egg—was significantly associated with illness (OR 11.4; 95% CI 1.3–139.4).

**Conclusions:**

This investigation demonstrates the value of analytical epidemiology in challenging early assumptions and identifying an unexpected exposure in the absence of food samples. Multidisciplinary coordination, while resource-intensive, enabled clear outcomes for affected individuals and regulators, and strengthened national surveillance intelligence.

